# OTU deubiquitinases as immune-circuit editors: from human immunopathology to therapeutic prioritization

**DOI:** 10.3389/fimmu.2026.1843621

**Published:** 2026-07-01

**Authors:** Yi Guo, Yue Song, Chenxin Wang, Rui Xiong, Xinyu Bao, Surui Lu, Wenhan Wu, Xijun Wang, Jiyuan Liao, Yongfen Bao, Lihua Qu, Zhiwei Rao, Qi Han

**Affiliations:** 1 School of Basic Medical Sciences, Xianning Medical College, Hubei University of Science and Technology, Xianning, China; 2 Hubei Key Laboratory of Environmental Risks and Related Diseases Precision Control, Hubei University of Science and Technology, Xianning, China; 3 School of Health Management, Xianning Vocational Technical College, Xianning, China; 4 School of Public Health and Nursing, Hubei University of Science and Technology, Xianning, China; 5 School of Stomatology and Ophthalmology, Xianning Medical College, Hubei University of Science and Technology, Xianning, China; 6 Department of Pharmacy, Xianning Central Hospital, The First Affiliated Hospital of Hubei University of Science and Technology, Xianning, China; 7 Department of Oncology, Xianning Central Hospital, The First Affiliated Hospital of Hubei University of Science and Technology, Xianning, China

**Keywords:** human immunopathology, immune-circuit editing, inflammatory signaling, OTU deubiquitinases, therapeutic prioritization, tumor immune remodeling, ubiquitin-chain selectivity

## Abstract

OTU deubiquitinases are linkage-sensitive ubiquitin editors that regulate immune circuits, human immunopathology, and emerging therapeutic opportunities. By remodeling K48-, K63-, and linear ubiquitin chains, they control protein stability, scaffold assembly, and signal amplitude across nuclear factor κB (NF-κB), and tumor-immune pathways. Individual OTUs have been linked to inflammation, infection, autoimmunity, metabolic dysfunction, and cancer, yet disease-by-disease descriptions often obscure their shared mechanistic logic. We therefore organize current evidence around chain selectivity, substrate context, and immune-circuit function. We examine innate inflammatory regulation, adaptive and tumor immunity, and the emerging therapeutic landscape, including covalent inhibitors, engineered binders, repurposed compounds, and induced-proximity platforms. We also distinguish catalytic from non-catalytic functions, define determinants of context dependence, and propose practical criteria for translational target ranking. This framework positions OTU enzymes not only as an isolated catalogue of disease factors, but also as a mechanistically coherent field centered on mechanism-guided target ranking and disease-specific biological context.

## 1 Introduction

Ubiquitination is a highly versatile post-translational modification that regulates protein turnover, signal assembly, intracellular trafficking, and stress adaptation. Its biological consequences are determined by the presence of ubiquitin conjugation. Other factors include the topology of ubiquitin chains, including degradative and non-degradative linkages that encode distinct signaling outputs (1–3). Deubiquitinases (DUBs) therefore function not merely as passive erasers of ubiquitin, but as active editors of signaling specificity.

Among the DUB families, ovarian tumor (OTU) domain-containing enzymes are of particular interest because many members exhibit substantial ubiquitin-chain selectivity and are deeply embedded in immune and inflammatory signaling networks (4–7). By editing K48-, K63-, and linear ubiquitin chains, OTU enzymes can terminate signaling by dismantling activating ubiquitin scaffolds. They also can preserve signaling by removing degradative ubiquitin marks from key effectors. This dual logic places the OTU family at a strategic interface between molecular signal decoding and disease biology.

Over the past decade, accumulating evidence has linked OTU enzymes to a broad disease spectrum, including inflammatory disorders, infection, autoimmunity, neuroinflammatory conditions, metabolic dysfunction, and cancer (5, 8–12). In oncology, OTU enzymes have emerged as regulators of tumor growth, invasion, stress adaptation, and immune escape. In immunology, they are increasingly recognized as linkage-sensitive rheostats that shape innate inflammatory amplitude, antiviral responses, and the balance between protective and pathological immunity. At the same time, the field is entering a translational phase, with early inhibitors, engineered binders, and repurposed compounds beginning to define the druggability of selected OTU targets (5, 8–12).

The literature is extensive but fragmented across individual enzymes and disease categories. Across these settings, OTU enzymes converge on a limited set of signaling programs, including nuclear factor κB (NF-κB), stimulator of interferon genes (STING), mitogen-activated protein kinase (MAPK), Wnt/β-catenin, cell death, and tumor–immune crosstalk. Organizing the field around these shared circuits provides a clearer basis for comparing context-dependent functions and therapeutic opportunities. Recent OTUD-focused syntheses have updated cancer and antiviral biology (13).

**Figure 1:**
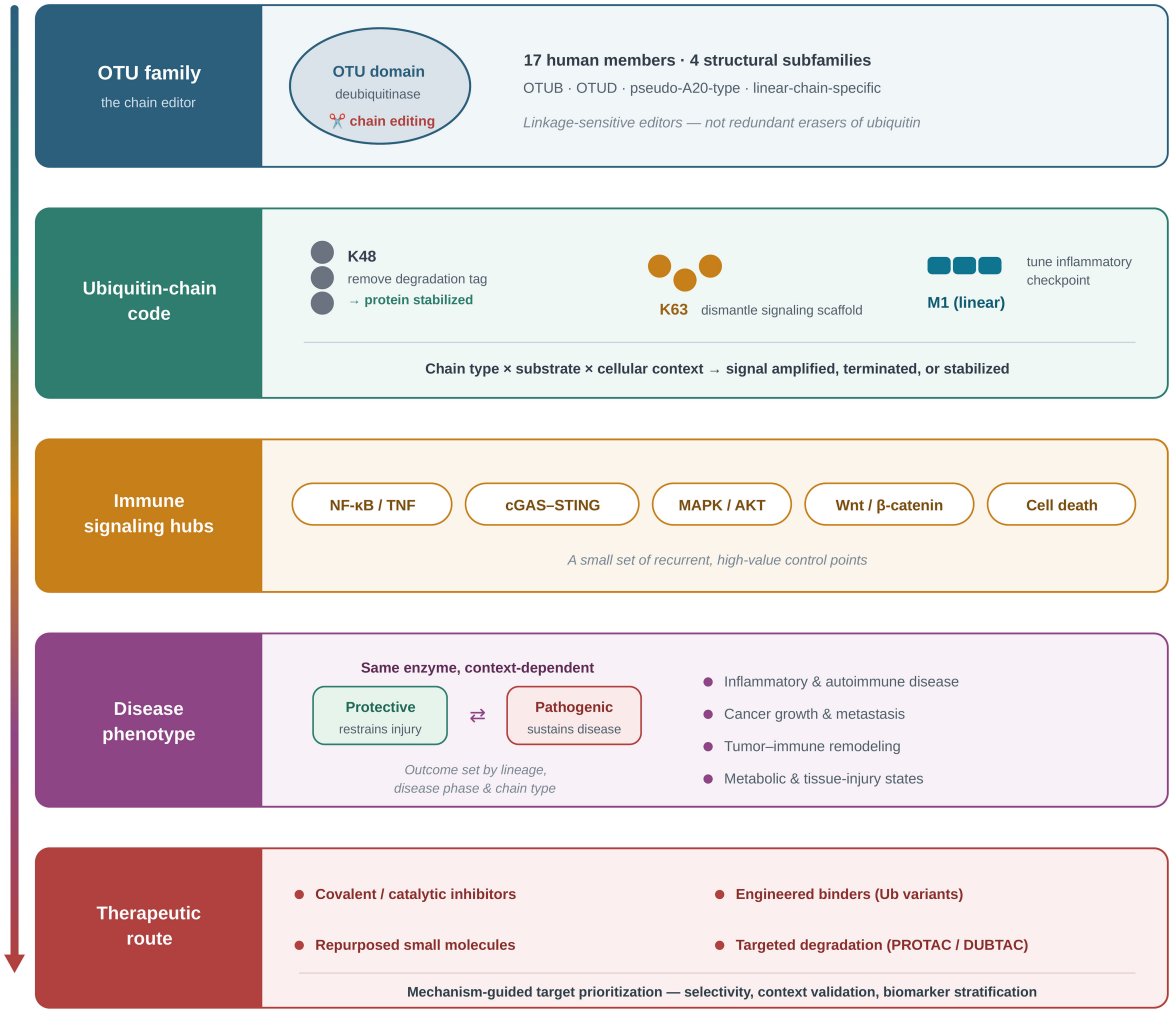
Mechanism-centered overview of OTU family biology. OTU enzymes are shown as linkage-sensitive ubiquitin editors that connect K48-, K63-, and linear-chain selectivity to immune signaling nodes, representative disease outputs, and therapeutic opportunities. The revised figure emphasizes conceptual flow rather than catalog-like listing.

The present review emphasizes immune-circuit editing, human immunopathology, and translational target ranking. We first examine how OTU enzymes decode ubiquitin-chain topology and achieve signal selectivity. We then discuss innate inflammatory regulation, adaptive and tumor immunity, and the main barriers to therapeutic translation. **Figure 1** presents the conceptual framework, and **Tables 1**, **2** summarize representative disease mechanisms and the inhibitor landscape, respectively.

**Table 1:** Representative disease-associated functions of OTU family members.

OTU member	Representative disease context	Principal substrate/node	Ubiquitin editing outcome	Representative pathway/program	Major biological effect	Key refs
OTUB1	Hepatocellular carcinoma	RACK1	Non-canonical deubiquitination; increased RACK1 stability	RACK1-associated oncogenic signaling	Promotes HCC growth and progression	(47)
OTUB1	Breast cancer	MYC	Deubiquitination; increased MYC stability	MYC/HK2-glycolysis	Promotes breast tumorigenesis	(42)
OTUB1	Breast cancer	CCN6	Non-canonical stabilization of CCN6	CCN6 tumor-suppressive signaling	Suppresses breast-cancer growth	(79)
OTUB1	Triple-negative breast cancer	ERRα	Reduced ERRα abundance after OTUB1 targeting	ERRα-driven metabolic/proliferative signaling	Suppresses TNBC growth	(80)
OTUB1	Colorectal cancer	SRPX2	Deubiquitination; increased SRPX2 stability	Autophagy/chemoresistance	Promotes chemoresistance and tumor progression	(52)
OTUB1	Gastric cancer	GPX4	Deubiquitination; increased GPX4 stability	Ferroptosis suppression	Inhibits ferroptosis and promotes metastasis	(81)
OTUB1	Multiple myeloma	c-Maf	K48 deubiquitination; increased c-Maf stability	c-Maf/MYC transcriptional program	Promotes myeloma growth	(82)
OTUB1	Lupus nephritis	SLC7A11	Deubiquitination; increased SLC7A11 stability	Ferroptosis/redox homeostasis	Restrains ferroptosis and protects renal injury	(83)
OTUB1	Rheumatoid arthritis	CCL5-associated immune signaling	Deubiquitination-associated chemokine regulation	NF-κB/chemokine signaling	Limits inflammatory-cell infiltration	(84)
OTUB1	Diabetic cardiomyopathy	YB-1	Reduced OTUB1 interaction enhances YB-1 phosphorylation	ERK/RSK-YB-1 signaling	Loss of OTUB1 restraint aggravates cardiomyopathy	(85)
OTUB2	Colorectal cancer	PKM2	Enhanced PKM2 activity	Glycolysis/metabolic reprogramming	Promotes CRC progression	(86)
OTUB2	Colorectal cancer	β-Catenin	Deubiquitination; increased β-catenin stability	Wnt/β-catenin	Promotes proliferation	(48)
OTUB2	Lung cancer	PD-L1	K48 deubiquitination; increased PD-L1 stability	Immune evasion/PD-L1 signaling	Promotes immune escape	(60)
OTUD1	Breast cancer	p53	Deubiquitination; increased p53 stability	p53 tumor-suppressive signaling	Inhibits tumor growth and promotes apoptosis	(87)
OTUD1	NSCLC	YAP1	Reduced nuclear YAP1 activity	Hippo/YAP; EGFR-TKI response	Enhances erlotinib sensitivity and suppresses growth	(88)
OTUD1	Inflammatory bowel disease	RIPK1	K63 deubiquitination; disrupted RIPK1-NEMO interaction	NF-κB	Alleviates intestinal inflammation	(37)
OTUD1	Acute lung injury	—	—	Inflammatory response/LPS-induced lung injury	OTUD1 deficiency alleviates inflammatory lung injury	(38)
YOD1	Triple-negative breast cancer	CDK1	Deubiquitination; increased CDK1 stability	Cell-cycle control	Promotes proliferation	(89)
YOD1	Colitis	RIPK2	Deubiquitination; increased RIPK2 stability	NOD2-mediated protective signaling	Sustains protective intestinal signaling	(90)
YOD1	Rheumatoid arthritis	YOD1-associated Hippo signaling	—	Hippo signaling	Contributes to inflammatory/proliferative signaling	(91)
YOD1	Acute promyelocytic leukemia	PML/RARα	YOD1 blockade drives oncoprotein degradation	PML/RARα leukemogenic program	YOD1 targeting suppresses leukemic growth	(71)
OTUD3	Hepatocellular carcinoma	ACTN4	Deubiquitination; increased ACTN4 stability	Cytoskeletal/metastatic signaling	Drives growth and metastasis	(50)
OTUD3	Breast cancer	p53	Deubiquitination; increased p53 stability	p53 tumor-suppressive signaling	Inhibits growth and promotes apoptosis	(92)
OTUD3	Breast cancer	PTEN	K48 deubiquitination; increased PTEN stability	PTEN/AKT tumor-suppressive signaling	Suppresses tumorigenesis	(8)
OTUD3	Colorectal cancer	YY1	Phosphorylation-dependent deubiquitination; increased YY1 stability	YY1 transcriptional program	Promotes CRC progression	(93)
OTUD3	Lung cancer	GRP78/OTUD3 axis	OTUD3 targeting destabilizes GRP78-associated proteostasis	Proteostasis/stress adaptation	Supports lung-cancer growth and druggability	(94, 95)
OTUD3	Metabolic homeostasis	PPARδ	K48 deubiquitination; increased PPARδ stability	Nutrient-stress metabolic adaptation	Maintains metabolic homeostasis	(78)
OTUD4	Breast cancer	CD73	Deubiquitination; increased CD73 stability	Adenosine/immune-suppressive signaling	Promotes immune suppression	(61)
OTUD4	Solid-tumor ferroptosis models	GPX4	Deubiquitination; increased GPX4 stability	Autophagy/ferroptosis	Suppresses ferroptosis and promotes tumor progression	(96)
OTUD5	Hepatocellular carcinoma	SLC38A1	Deubiquitination; increased SLC38A1 stability	Amino-acid transport/growth signaling	Promotes HCC growth	(97)
OTUD5	Triple-negative breast cancer	YAP	Deubiquitination; increased YAP stability	Macrophage M2 polarization/Hippo-YAP	Favors TNBC progression via M2 polarization	(62)
OTUD5	Diabetic kidney disease	TAK1	K63 deubiquitination; reduced TAK1 phosphorylation	TAK1-MAPK inflammatory signaling	Alleviates renal inflammation and injury	(40)
OTUD6A	Breast cancer	TopBP1	K48 deubiquitination; increased TopBP1 stability	DNA-damage response	Promotes progression and therapy resistance	(98)
OTUD6A	Colitis	NLRP3	K48 deubiquitination; increased NLRP3 stability	Inflammasome signaling	Promotes intestinal inflammation and colitis	(99)
OTUD6A	Prostate cancer	c-Myc	Deubiquitination; increased c-Myc stability	c-Myc oncogenic signaling	Promotes tumorigenesis	(100)
OTUD6B	Hepatocellular carcinoma	pVHL	Deubiquitination-associated pVHL stabilization	pVHL/HIF-1α axis	Suppresses HCC metastasis	(41)
OTUD6B	Multiple myeloma	LIN28B	Deubiquitination; increased LIN28B stability	LIN28B-MYC axis	Promotes proliferative state	(49)
A20	Inflammatory bowel disease	ABIN3/RIPK3 complex	Restricted RIPK3 ubiquitination via ABIN3-A20 axis	Necroptosis/intestinal inflammation	Alleviates intestinal inflammation	(101)
A20	Acute lung injury	STAT3/A20/ASK1 axis	—	STAT3/A20/ASK1 inflammatory signaling	Attenuates acute lung injury	(102)
OTULIN	OTULIN-related autoinflammatory syndrome (ORAS)	Met1-linked (linear) ubiquitin chains on LUBAC-associated substrates	Hydrolysis of linear ubiquitin chains; restraint of LUBAC-dependent NF-κB activation	LUBAC/linear ubiquitin/NF-κB homeostasis	Loss-of-function causes systemic sterile inflammation, recurrent fever, and multi-organ inflammatory damage	(9, 28)
OTULIN	Liver inflammation and fibrosis	Met1-linked ubiquitin chains; hepatocyte death-associated signaling	Linear chain editing; suppression of hepatocyte apoptosis and inflammatory amplification	TNF-dependent cell death/NF-κB/fibrotic remodeling	OTULIN deficiency promotes liver cell death, chronic inflammation, fibrosis, and progression toward hepatocellular carcinoma	(27)
OTUD7A	Ewing sarcoma	EWS-FLI1	Deubiquitination-associated stabilization of EWS-FLI1	EWS-FLI1 oncogenic program	Supports Ewing sarcoma growth	(103)
OTUD7B	Breast cancer	LSD1	K63 deubiquitination; increased LSD1 stability	Epigenetic/metastatic program	Promotes metastasis	(104)
OTUD7B	Lung cancer	TRAF3	Deubiquitination; stabilized TRAF3	TRAIL receptor complex II/invasive signaling	Suppresses invasion and migration	(53)
ZRANB1	Hepatocellular carcinoma	LOXL2	Deubiquitination-associated activation/stabilization	SP1-LOXL2 axis	Drives HCC progression	(105)
OTUD1	Alzheimer’s disease/microglial neuroinflammation	C/EBPβ	K48 deubiquitination; increased C/EBPβ stability	Microglial inflammatory transcription program	Promotes neuroinflammation and aggravates AD pathology	(33)
OTUD5	Sepsis-induced acute lung injury	GBP2–OTUD5–GPX4 axis	GBP2 binding promotes GPX4 ubiquitination/degradation	Endothelial ferroptosis/barrier injury	Drives endothelial ferroptosis and lung injury	(34)
OTUD4	Inflammation-driven NSCLC	TAK1/TAB3 signalosome	K63 deubiquitination; reduced TAK1/TAB3 signaling	TNF-induced NF-κB/chronic inflammation	Suppresses inflammation-driven oncogenesis	(56)
OTUD1	NSCLC	RAD23B/XPC	K63 deubiquitination coupled to PRKN-dependent degradation	Nucleotide-excision repair/platinum response	Enhances cisplatin sensitivity	(54)
OTUB1	Colorectal cancer	GPX4	Deubiquitination; increased GPX4 stability	Ferroptosis suppression	Promotes CRC progression	(57)
OTUB1	Metastatic tumor models	PP1α	Deubiquitination; increased PP1α stability	ERK1/2-mediated anoikis resistance	Promotes metastasis and anoikis resistance	(58)

Mechanistically supported examples are organized by disease context, principal substrate or node, ubiquitin-editing outcome, representative pathway or program, biological effect, and key references.

GPX4, glutathione peroxidase 4; NF-κB, nuclear factor κB; NSCLC, non-small cell lung cancer.

**Table 2:** Emerging therapeutic strategies targeting OTU enzymes.

Target OTU	Compound/strategy	Modality	Evidence level	Disease context	Selectivity/cross-reactivity and key limitation	Key refs
OTUB1	Ailanthone	Natural-product OTUB1 inhibitor	Cellular and mouse preclinical	Triple-negative breast cancer/ERRα axis	C91-dependent target engagement is supported, but systematic profiling across the DUB family has not been reported; broader selectivity and medicinal-chemistry optimization remain necessary.	(80)
OTUB1	Erianin	OTUB1-targeting degradation-inducing natural product	Cellular and mouse preclinical	Esophageal squamous cell carcinoma metastasis	OTUB1 targeting and degradation are supported, but broad DUB counter-screening and pharmacokinetic characterization remain unresolved.	(106)
OTUB1	Compound 61 (OTUB1/USP8 dual inhibitor)	Covalent dual-target inhibitor	Biochemical, cellular, and mouse preclinical	NSCLC and proteostasis-driven oncology models	Dual OTUB1/USP8 activity is intentional; the compound is not OTUB1-selective, and broader off-target liabilities require definition.	(66)
OTUB2	OTUB2-IN-1	Direct catalytic inhibitor	Biochemical, cellular, and syngeneic-mouse preclinical	PD-L1-driven immune-evasion models; lung cancer-relevant contexts	Direct OTUB2 inhibition is supported, but comprehensive cross-DUB profiling, pharmacokinetics, and independent validation remain limited.	(60)
OTUD3	Rolapitant	Repurposed small molecule/direct OTUD3 inhibitor	Cellular and xenograft preclinical	Lung cancer/OTUD3-GRP78 axis	OTUD3 binding and functional inhibition are supported; systematic profiling against other DUBs has not been reported.	(95)
OTUB2	LN5P45	Improved covalent active-site inhibitor	Biochemical and cellular target-engagement stage	OTUB2 target-validation in cancer biology	Chemical target engagement is strong, but comprehensive DUB-family selectivity and *in vivo* disease positioning remain incomplete.	(10)
OTUD3	Rupatadine	Repurposed small molecule	Cellular and *in vivo* preclinical	Diffuse large B-cell lymphoma/PD-L1 and MYL12A axis	Competitive OTUD3 binding is supported; broader DUB counter-screening and the translational window require further validation.	(107)
YOD1	G5 (ubiquitin isopeptidase inhibitor I)	Small-molecule DUB inhibitor repurposed as a YOD1-directed lead	Cellular, patient-derived, and mouse preclinical	Acute promyelocytic leukemia, including drug-resistant models	G5 is a broad DUB-active compound and should not be interpreted as YOD1-selective.	(71)
OTUD5	No validated direct small-molecule inhibitor yet	Target-biology only	Preclinical target-biology evidence	OTUD5-GPX4 ferroptosis axis	Not applicable: no validated OTUD5-directed ligand is available; this entry reflects target biology only.	(108)
OTUD7A	No validated direct small-molecule entry retained here	Target-biology only	Biology supported; pharmacologic entry not retained	Ewing sarcoma/EWS-FLI1 stability	Not applicable: no validated OTUD7A-directed ligand is retained; this entry reflects target biology only.	(103)
OTUD7B	7Bi	AI-aided small-molecule inhibitor	Biochemical and cellular preclinical	NSCLC and leukemia models	Initial activity is supported, but comprehensive DUB-panel profiling, direct-binding validation, pharmacokinetics, and medicinal-chemistry optimization remain incomplete.	(69)
Multiple/future platform	OTUD1 UbVOD.1; DUBTAC/PRO-DUBTAC/induced-proximity concepts	Protein-based inhibitor or conceptual platform	*In vitro*/cellular tool to review-stage concept	OTUD1 target validation; broader biomarker-selected tumor or immune contexts	Selectivity depends on the recruited DUB ligand and productive ternary-complex formation; no clinically deployable OTU-directed platform is established.	(72–75)

Entries are organized by target, modality, evidence level, disease context, selectivity/cross-reactivity evidence, key limitation, and supporting reference. When broad DUB-panel counter-screening, chemoproteomic profiling, or orthogonal cellular target-engagement data have not been reported, the compound is described as a proof-of-concept or target-validation tool rather than a definitive OTU-selective therapeutic.

AI, artificial intelligence; APL, acute promyelocytic leukemia; DLBCL, diffuse large B-cell lymphoma; DUB, deubiquitinase; DUBTAC, deubiquitinase-targeting chimera; ERRα, estrogen-related receptor alpha; EWS-FLI1, Ewing sarcoma breakpoint region 1–Friend leukemia integration 1 fusion oncoprotein; GPX4, glutathione peroxidase 4; GRP78, glucose-regulated protein 78; HCC, hepatocellular carcinoma; NSCLC, non-small cell lung cancer; OTU, ovarian tumor; PD-L1, programmed death-ligand 1; PK, pharmacokinetics; PROTAC, proteolysis-targeting chimera; TNBC, triple-negative breast cancer; USP, ubiquitin-specific protease.

## 2 Ubiquitin chain editing and signal selectivity of OTU enzymes

**Figure 2:**
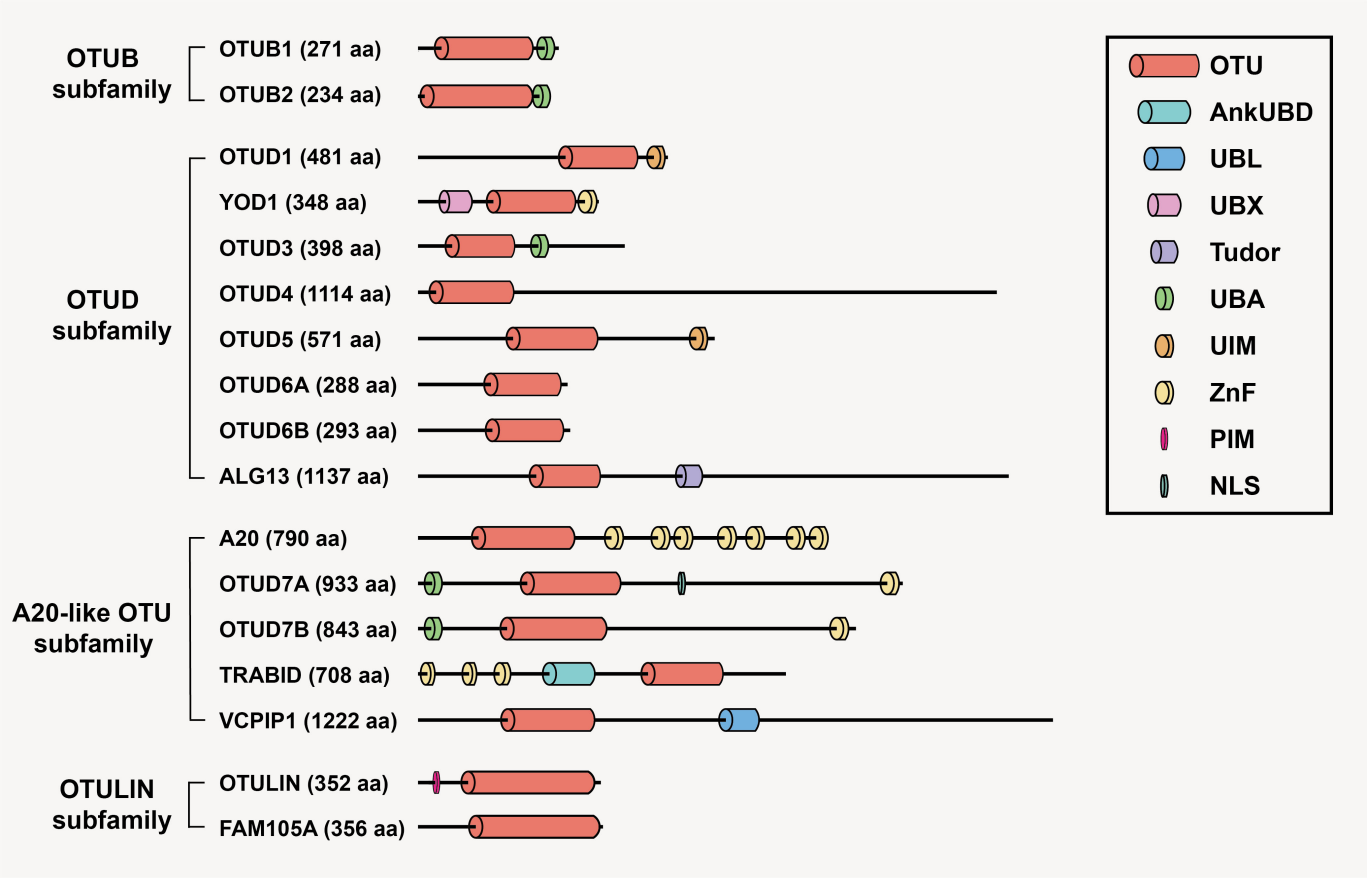
Domain architecture and subfamily organization of human OTU deubiquitinases. OTUB, OTUD, pseudo-A20-type, and linear-chain-specific OTUs differ in accessory domains, linkage preference, and subcellular targeting, providing a structural basis for pathway selectivity and non-redundant function.

A defining property of the OTU family is that its members are not functionally redundant. Instead, they differ in catalytic architecture, accessory domains, substrate-recognition modes, and ubiquitin-linkage preference (4, 5, 14). This biochemical diversity enables OTU enzymes to function as pathway-selective regulators rather than generic suppressors of ubiquitination. In biological terms, OTU-dependent deubiquitination can either dampen signaling by removing non-degradative activating chains or stabilize signaling proteins by counteracting K48-linked proteasomal targeting. The structural and domain-level heterogeneity of OTU family members is summarized in **Figure 2**.

Related catalytic cores do not imply interchangeable functions. Non-redundancy is imposed by linkage preference, stimulus-dependent recruitment, subcellular localization, lineage-specific expression, and substrate access. These features place individual OTUs in distinct molecular neighborhoods and time windows, limiting functional interference even when catalytic chemistry is shared (4, 5). This distinction is central to interpreting the seemingly divergent functions of OTU enzymes across diseases. When an OTU removes K48-linked ubiquitin chains from an oncogenic transcription factor, stress-response protein, or signaling intermediate, it prolongs protein half-life and may amplify disease-driving programs. By contrast, when it removes K63- or linear ubiquitin chains from receptor-associated scaffold proteins or inflammatory signaling complexes, it can attenuate signal propagation and restore homeostatic control (2, 4, 7, 15). The biological outcome of OTU activity is therefore determined by at least three interacting parameters: chain type, substrate identity, and cellular context.

At the systems level, a relatively small set of signaling axes repeatedly emerges as OTU-sensitive. These include NF-κB-associated inflammatory complexes, STING-dependent innate immune signaling, MAPK and AKT pathways, Wnt/β-catenin signaling, cell death regulators, and proteins that shape redox adaptation or immune checkpoint biology (7, 16–19). This recurrent convergence suggests that OTU enzymes should not be conceptualized as isolated enzyme–substrate pairs, but as a family of ubiquitin editors that repeatedly regulate a limited number of biologically decisive signaling hubs.

Apparent contradictions usually reflect context rather than inconsistency. An OTU that terminates receptor-proximal inflammatory signaling in an immune cell may stabilize an oncogenic or immune-evasive protein in a stressed tumor cell. The relevant unit of analysis is therefore the enzyme–substrate–cell-state combination, not the enzyme alone. Thus, OTU family unity lies in a shared logic of linkage-sensitive editing, whereas biological output is determined by recruitment and substrate context. This framework underpins the immune and cancer sections below.

## 3 OTUs in innate immune and inflammatory programs

The most conceptually mature area of OTU biology lies in innate immune and inflammatory signaling. Multiple family members function as regulators of receptor-proximal ubiquitin editing. They control signal amplitude, duration, and resolution during inflammatory activation (16, 20, 21). OTU enzymes often behave as rheostats that calibrate the balance between protective host defense and pathological tissue injury.

A20 remains the canonical example. A20 is a multifunctional regulator with both deubiquitinase-related and ubiquitin-binding activities. It is central to the containment of excessive inflammatory signaling, particularly along NF-κB-associated pathways (22–25). Its importance extends beyond basic signaling biology: maintaining immune tolerance and preventing persistent inflammatory damage. A20 represents one of the demonstrations that defective ubiquitin editing can translate into systemic immune dysregulation. Recent clinical synthesis extends the spectrum of A20 haploinsufficiency (HA20) toward lupus-like autoimmunity. This emphasizes that A20 deficiency is relevant not only to autoinflammatory phenotypes but also to monogenic lupus-spectrum disease with renal, cutaneous, and systemic manifestations (26).

OTU deubiquitinase with linear linkage specificity (OTULIN) provides a complementary paradigm by controlling linear ubiquitin homeostasis and thereby safeguarding inflammatory balance at the level of linear ubiquitin chain assembly complex (LUBAC)-associated signaling (6, 7, 9, 27, 28). While A20 is mainly related to inflammatory restraint, OTULIN highlights the importance of linkage-specific editing in the quality of downstream immune output. Together, A20 and OTULIN establish a central principle: OTU enzymes are not merely enzymes acting downstream of inflammation, but active architects of inflammatory signal fidelity. Recent data also expand OTULIN-associated disease beyond canonical OTULIN-related autoinflammatory syndrome (ORAS), showing that heterozygous OTULIN haploinsufficiency can underlie trigger-dependent necrosis. These findings reinforce the clinical importance of linear-ubiquitin control in human inflammatory pathology (29).

Beyond these archetypal regulators, a broader set of OTU enzymes participates in more context-dependent innate immune programs. OTUD1 and OTUD5, for example, have been implicated in inflammatory activation, tissue injury responses, and stress-responsive signaling. YOD1 and selected OTUB family members appear to fine-tune disease-associated inflammatory states (16–19, 21, 30–32). OTU enzymes frequently operate at signaling bottlenecks where immune activation intersects with cell survival, barrier function, and tissue remodeling. Recent work has further extended OTUD1 biology to neuroinflammation. OTUD1 deubiquitinates and stabilizes C/EBPβ in microglia, amplifying inflammatory responses and worsening Alzheimer-related pathology. OTUD5-linked biology has expanded beyond interferon-centered models. A macrophage extracellular vesicle (EV)–GBP2-OTUD5-glutathione peroxidase 4 (GPX4) axis has been shown to drive endothelial ferroptosis and barrier injury in sepsis-induced acute lung injury (33, 34). OTUD5 illustrates how regulatory plasticity can create therapeutic entry points beyond direct inhibition. Phosphorylation of Ser177 is required for catalytic activation, while mTOR-dependent phosphorylation connects OTUD5 activity to nutrient-responsive mTORC1/2 signaling. Recruitment to specific complexes further changes substrate access. Upstream kinases, interaction surfaces, and turnover pathways may therefore be tractable even when a selective OTUD5 inhibitor is unavailable (35, 36).

Context dependence is determined by the substrate available to the OTU, the cell lineage and subcellular compartment, the intensity and duration of the initiating stimulus, and the surrounding metabolic or redox state (24, 37–40). During acute Tumor necrosis factor (TNF) or Toll-like receptor (TLR) signaling, A20 recruitment to ubiquitin-rich receptor complexes promotes resolution and protects tissue. In chronic inflammation or a hypoxic, therapy-stressed tumor, the dominant substrate pool may instead favor stabilization of survival, repair, or immune-evasion proteins. OTUD1 similarly restrains Receptor-interacting serine/threonine-protein kinase 1 (RIPK1)–NF-κB signaling in colitis yet can increase platinum sensitivity by destabilizing the RAD23 homolog B (RAD23B)/XPC repair complex. Protective or pathogenic labels are therefore meaningful only when cell state, substrate, and disease phase are specified (22–25).

**Figure 3:**
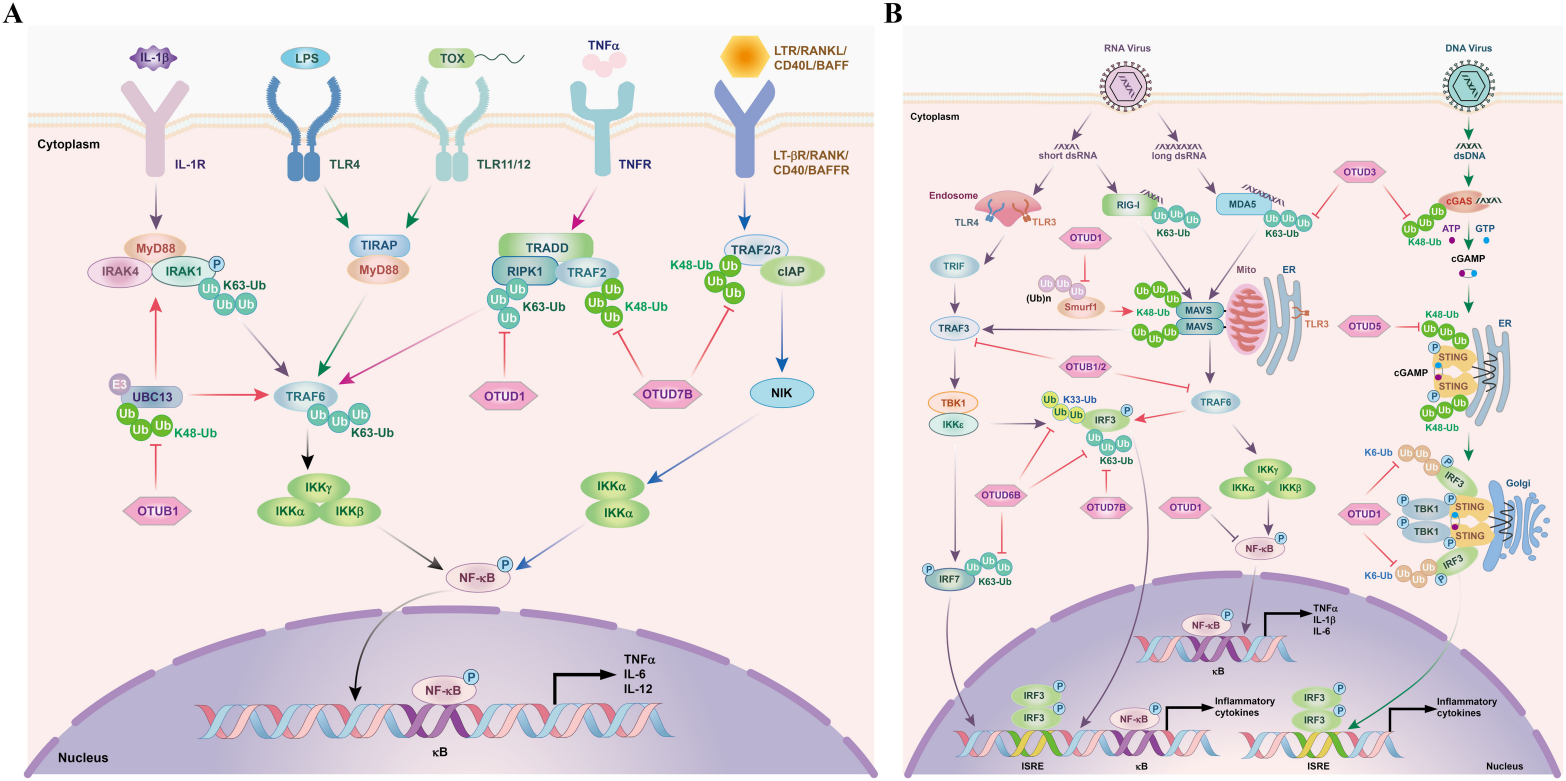
OTU-mediated control of innate inflammatory and antiviral signaling. **(A)** summarizes OTU-dependent control of TLR-, TNF-, and IL-1R-associated inflammatory signaling. **(B)** highlights regulation of RIG-I/MDA5/MAVS and cGAS-STING antiviral pathways. Together, the panels emphasize OTU positioning at proximal signaling bottlenecks.

A similar principle applies to acute inflammatory injury, antiviral signaling, and inflammasome-associated contexts. Emerging work indicates that OTU-mediated ubiquitin editing may influence not only cytokine output, but also cell fate decisions, including survival, apoptosis-related signaling, and inflammatory tissue remodeling. These observations reinforce the notion that OTU enzymes are best understood as signal-integrating regulators rather than isolated inflammatory modifiers. Representative OTU-mediated control of canonical inflammatory and antiviral pathways is illustrated in **Figure 3** (16–19, 21, 30). OTU enzymes therefore occupy proximal control points that couple innate signaling to cell fate and tissue injury. Representative immune and disease associations are summarized in **Table 1**.

Additional lineage-specific examples are summarized in **Supplementary Figure 1**. It highlights OTU-dependent signaling in Natural killer (NK) cells, neutrophils, mast cells, dendritic cells, and stem-cell-associated inflammatory niches. Representative mechanistic evidence for these lineage-specific roles includes A20-dependent regulation of mast cell and dendritic cell activation thresholds (22–25), OTULIN-mediated control of Linear ubiquitin chain assembly complex (LUBAC)-associated signaling in myeloid lineages (6, 7, 9, 28), and OTUD family contributions to innate immune cell survival and effector function (31, 32). Representative non-neoplastic disease contexts are summarized in **Supplementary Figure 2**, which links recurrent OTU-dependent mechanisms to intestinal inflammation, acute lung injury, rheumatoid arthritis, systemic lupus erythematosus, neuroinflammation, and metabolic disease (24, 25, 28, 31, 32, 37–40).

## 4 OTUs in adaptive immunity and tumor immune remodeling

The contribution of OTU enzymes to cancer is most productively understood through common malignant programs. In solid and hematologic malignancies, OTU enzymes mainly influence three processes: maintenance of proliferative and survival signaling, promotion of invasion and phenotypic plasticity, and modulation of tumor–immune interactions (41–46).

First, several OTU family members stabilize proteins that support tumor growth and survival. OTU domain-containing ubiquitin aldehyde-binding proteins (OTUB) and other members have been linked to the preservation of oncogenic effectors, transcriptional regulators, metabolic proteins, or pathway intermediates that collectively sustain proliferation and stress tolerance (42, 47–49). Although the specific substrates differ across models, the mechanistic logic is consistent: OTU-mediated removal of degradative ubiquitin chains increases protein stability and reinforces signaling output. These have been reported in breast, lung, colorectal, liver, and hematologic cancers (**Table 1**).

Second, OTU enzymes contribute to metastatic behavior and treatment adaptation. By stabilizing proteins involved in epithelial–mesenchymal transition, extracellular matrix remodeling, redox homeostasis, and cytoskeletal plasticity, they can promote invasion, migration, and resistance to hostile microenvironmental conditions (50–55). In this setting, OTU enzymes should not be viewed solely as growth regulators. Rather, they function as facilitators of tumor adaptability, enabling malignant cells to tolerate therapeutic stress, sustain invasive phenotypes, or exploit inflammatory niches within the tumor microenvironment. In non-small cell lung cancer (NSCLC), OTUD1 enhances cisplatin sensitivity by destabilizing the RAD23B/XPC nucleotide-excision-repair complex, directly linking OTU biology to platinum response. OTUD4 has likewise emerged as a K63-linkage-selective suppressor of the Transforming growth factor-beta-activated kinase 1 (TAK1) signalosome that restrains TNF-induced NF-κB signaling and inflammation-driven oncogenesis. The same period also expanded OTUB1 biology in cancer, linking OTUB1 to ferroptosis-associated colorectal cancer progression through GPX4 stabilization and to anoikis resistance/metastasis through Protein phosphatase 1 alpha (PP1α) stabilization (54, 56–58).

**Figure 4:**
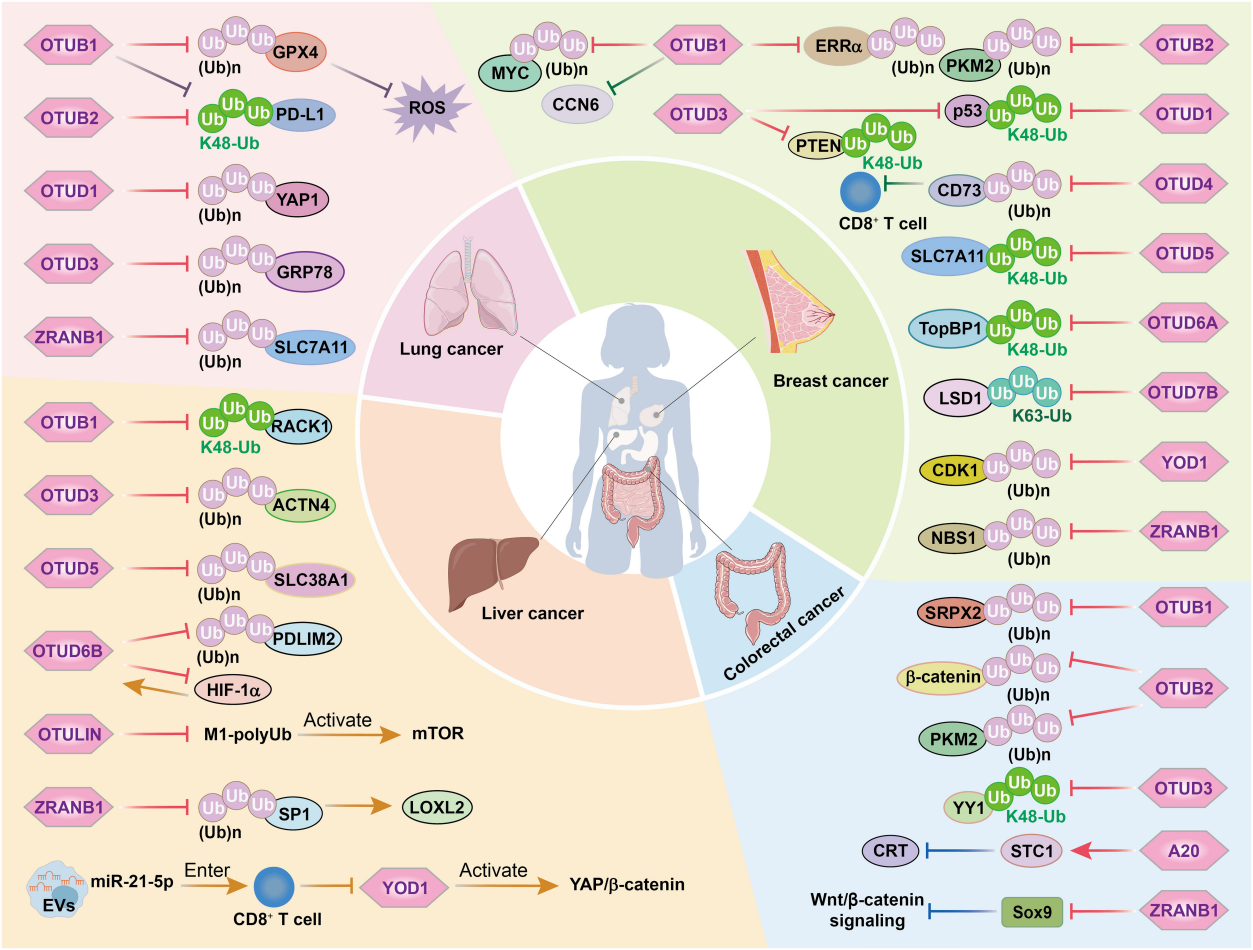
Representative OTU-dependent oncogenic programs across major cancer contexts. Mechanistically supported examples from major cancer contexts are organized into three recurring programs: stabilization of oncogenic or stress-response effectors, promotion of invasive plasticity, and modulation of tumor-immune interactions.

Third, OTU enzymes appear to intersect with tumor immunity. Available evidence suggests that they can influence inflammatory tone within tumors, the stability of immune-relevant proteins, antigen-processing-associated pathways, and checkpoint-related signaling (59–62). This places OTU enzymes at the interface between cell-intrinsic oncogenic signaling and cell-extrinsic immune remodeling. This interface opens the possibility that OTU targeting could reshape tumor immunogenicity or sensitize tumors to immunotherapy. Representative OTU-dependent oncogenic programs across major cancer contexts are summarized in **Figure 4**.

OTU-dependent immune remodeling operates at several levels. OTUB2-mediated stabilization of programmed death-ligand 1 (PD-L1) directly weakens cytotoxic T-cell activity, whereas OTUD4/CD73 signaling and OTUD5-driven M2 polarization create adenosine-rich and myeloid-suppressive niches that favor T-cell exhaustion. OTU control of NF-κB, STING, and interferon signaling may also alter dendritic-cell maturation, inflammatory licensing, and the transcriptional environment required for major histocompatibility complex (MHC) class I antigen presentation. Direct evidence that individual OTUs regulate peptide loading or MHC-I turnover remains limited, and this distinction should be preserved (60, 63–65). These microenvironmental effects are relevant to checkpoint blockade and chimeric antigen receptor T-cell (CAR-T) therapy because persistent antigen exposure, inhibitory ligands, suppressive myeloid cells, and metabolic stress constrain T-cell fitness. Direct evidence that OTU manipulation improves CAR signaling, CAR-T persistence, or exhaustion reversal is currently sparse. OTU-directed approaches are therefore better viewed as candidate tumor-microenvironment conditioning strategies than as established CAR-T engineering targets (60, 63–65).

**Figure 5:**
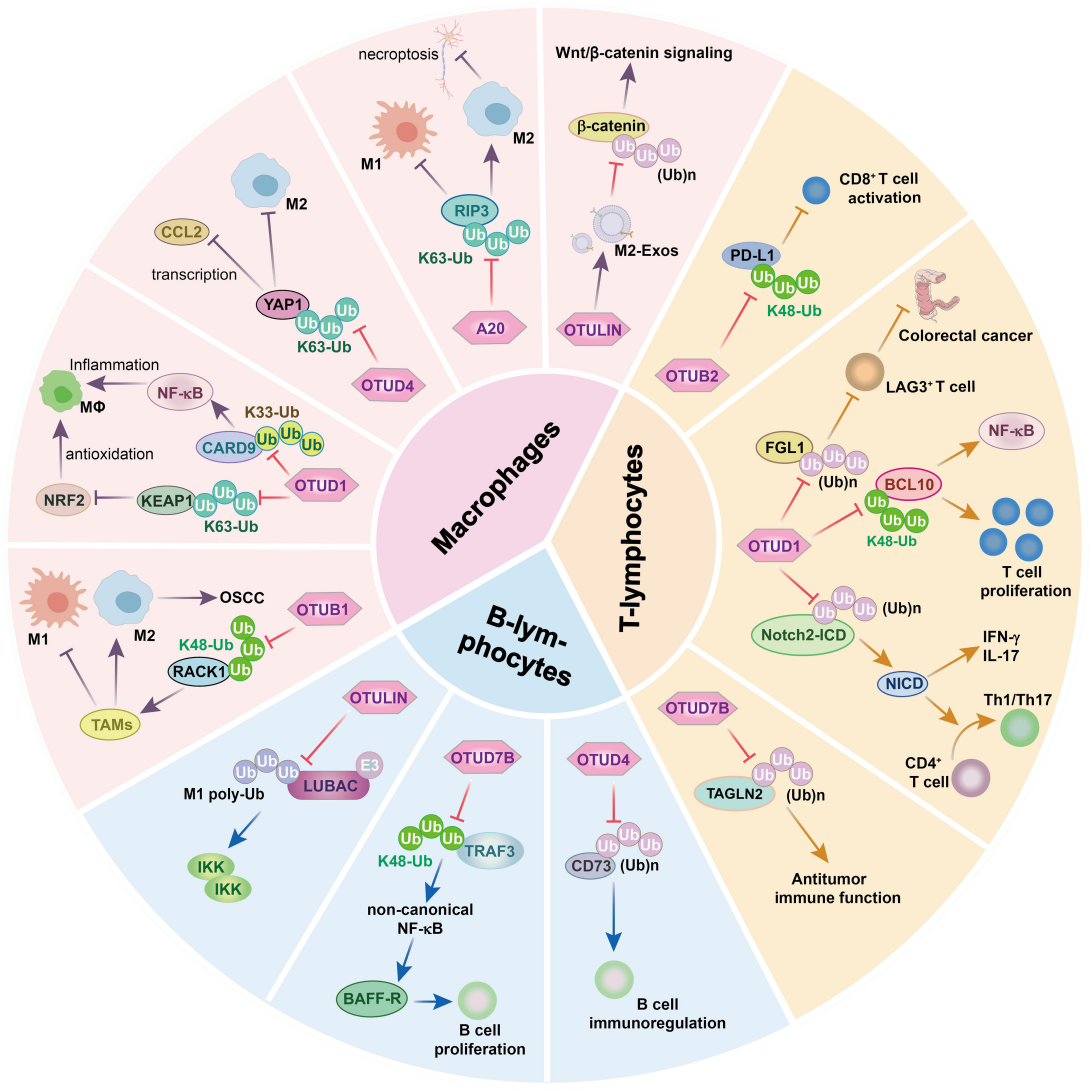
OTU functions in immune compartments relevant to tumor-associated immunity. The figure summarizes OTU-regulated signaling across macrophages, B cells, and T cells, emphasizing checkpoint stability, inflammatory polarization, and pathways linked to T-cell activation or exhaustion. This immune-compartment view complements the tumor-cell-intrinsic programs shown in **Figure 4**.

Beyond their tumor-intrinsic functions, OTU enzymes also operate across multiple immune-cell compartments that shape tumor-associated immunity. As shown in **Figure 5**, OTU family members regulate macrophage inflammatory polarization, B-cell signaling-associated survival programs, and T-cell activation or exhaustion-related pathways through control of nodes such as PD-L1, B-cell lymphoma/leukemia 10 (BCL10), RIPK3, Tumor necrosis factor receptor-associated factor 3 (TRAF3), and LUBAC-associated signaling. This cell-centered view extends the program-based framework outlined above and underscores that OTU biology in cancer should not be reduced to malignant cell-autonomous signaling alone. Instead, OTU-dependent ubiquitin editing acts across both tumor and immune compartments, thereby providing a mechanistic basis for tumor immune remodeling and potential combinatorial therapeutic intervention (59–62).

## 5 Druggability and therapeutic targeting of OTU enzymes

The translational landscape of OTU biology has evolved from conceptual interest to early-stage pharmacologic feasibility. Although no OTU-directed therapy has yet entered routine clinical application, several lines of evidence indicate that selected family members are tractable targets for small molecules, covalent inhibitors, engineered binders, and repurposed drugs (10–12, 46, 66–68).

Current strategies fall into four categories: direct small-molecule or covalent inhibitors, engineered binders such as ubiquitin variants, repurposed drugs, and induced-proximity platforms. Direct inhibitors and binders provide target-validation tools, whereas repurposed compounds offer a faster translational entry but often have uncertain selectivity (10, 46, 60, 66, 67, 69–74). DUBTAC and PRO-DUBTAC approaches remain concept-stage strategies that recruit a DUB to stabilize a protein of interest. Recent PRO-DUBTAC work demonstrates OTUB1-dependent stabilization of selected tumor-suppressive E3 ligases, but it does not yet establish a clinically deployable OTU-selective platform (75).

Therapeutically relevant OTU functions are not always catalytic. A20 is recruited to ubiquitin-rich signaling complexes through zinc-finger-dependent interactions and uses scaffold and ubiquitin-binding functions to organize signal termination. For such proteins, active-site inhibition may not reproduce genetic loss and may leave non-catalytic signaling intact. Disrupting ubiquitin-binding surfaces, recruitment interfaces, or disease-specific protein-protein interactions may therefore be more effective and potentially more selective than catalytic blockade alone (22, 23, 76). The inhibitor landscape remains predominantly preclinical. Most compounds are chemical probes or early leads rather than clinically mature candidates. **Table 2** shows target-biology entries, biochemical or cellular probes, *in vivo* leads, and concept-stage platforms. To make the concept of translational target ranking more explicit, we propose five practical criteria for prioritizing OTU targets in future development:

- human genetic or clinicopathologic evidence linking the OTU to a defined disease phenotype;
- a reproducible mechanistic axis with a tractable substrate, signaling node, or immune-circuit function;
- demonstrable *in vivo* phenotypic impact in disease-relevant models;
- evidence of target engagement together with at least preliminary selectivity or cross-reactivity profiling;
- a biomarker-defined context in which therapeutic benefit is likely to outweigh disruption of protective homeostatic roles.

Three barriers remain (10, 12, 67, 69, 70, 72–74). First, selectivity is often incompletely established because conserved DUB catalytic architecture permits cross-reactivity, while many studies do not report broad biochemical panels, chemoproteomic profiling, or orthogonal cellular target-engagement assays. **Table 2** therefore distinguishes compounds with limited or unreported cross-DUB profiling from better-characterized target-validation tools. Second, context dependence requires testing in disease-relevant immune and tissue compartments. Third, biomarker frameworks that identify likely responders remain scarce. Induced-proximity platforms add further constraints. Productive activity depends on ternary-complex geometry and cooperativity, and excessive concentrations of a bivalent compound can favor nonproductive binary complexes, producing a high-dose hook effect. These properties require concentration-response studies and direct assessment of ternary-complex formation rather than reliance on binary binding alone (73, 77).

## 6 Perspectives: from disease catalogs to mechanism-based translation

A central problem in the current OTU literature is structural rather than informational. The field has generated substantial mechanistic detail. But it is often distributed across isolated disease reports and enzyme-specific narratives, making it difficult to identify the recurrent principles that matter most for translation. Our synthesis suggests that OTU enzymes are best conceptualized as linkage-sensitive regulators positioned at the intersection of immune signaling, tissue adaptation, and disease progression (5, 11, 46, 68). The publication of multiple 2025–2026 OTU reviews raise the novelty threshold for broad family-wide summaries. They also increase the value of reviews that integrate recent human genetics, immune-circuit logic, and translational prioritization into a single framework (13).

This perspective yields two practical conclusions. First, the real value of OTU enzymes lies not in the implicated diseases, but in a limited set of biologically decisive pathways. These include NF-κB-centered inflammatory control, linear ubiquitin homeostasis, innate immune signal assembly, malignant protein stabilization, and tumor-immune crosstalk. Second, the translational relevance of OTU enzymes depends on context-specific prioritization. It is no longer sufficient to designate an OTU as broadly pathogenic or broadly protective. What matters is how that enzyme edits particular ubiquitin topologies in defined cellular states and whether such editing is therapeutically actionable (7, 9, 11, 19, 44, 46, 78). Taken together, linkage selectivity, recurrent pathway convergence, and increasingly tractable chemical or engineered intervention argue that the OTU family merits consideration as a distinct translational field rather than a loose collection of DUB case studies.

Context-specific prioritization is particularly important in oncology, where OTU-directed therapy may be most useful in combination with checkpoint blockade, cellular therapy, or pathway-specific agents. In inflammatory disease, the same principle requires separating OTUs that resolve injury from those that sustain pathological signaling.

Future work should therefore proceed along four parallel lines: deeper structural resolution of targetable OTU surfaces, more systematic substrate and chain-topology mapping, better integration of immune phenotyping with disease models, and biomarker-guided selection of disease contexts most likely to respond to OTU perturbation (5, 11, 12, 68, 73, 74). Such an agenda would move the field from descriptive enzymology toward clinically meaningful pathway engineering.

## 7 Conclusion

The OTU deubiquitinase family has emerged as a functionally specialized group of ubiquitin editors that link chain selectivity to immune control, inflammatory balance, tumor progression, and therapeutic opportunity. Although individual OTU enzymes differ substantially in substrate usage and biological context, they repeatedly converge on a limited number of signaling programs that govern inflammation, cell fate, stress adaptation, and tumor–host interaction (7, 19, 44, 78).

Current evidence supports two parallel conclusions. First, OTU enzymes are indispensable regulators of immune and disease biology whose actions are deeply context dependent. Second, their druggability is now sufficiently established to justify more systematic therapeutic development, provided that future work addresses selectivity, context-specific target validation, and biomarker-guided stratification (10, 67, 69, 70, 72). A mechanism-centered framework, rather than a catalog of isolated disease associations, therefore offers a productive path for translating OTU biology into clinically meaningful intervention. The field is now moving from descriptive enzymology toward disease-specific, mechanism-guided intervention.

## Supplementary material

Supplementary Figure 1 Cell-type-specific OTU-dependent regulation in innate immune and stromal-associated contexts. Representative lineage-specific OTU functions in NK cells, neutrophils, mast cells, dendritic cells, and stem-cell-related inflammatory niches are shown to emphasize cell-state-dependent control of inflammatory signaling thresholds.

Supplementary Figure 2 Representative non-neoplastic disease contexts associated with OTU dysregulation. Representative non-neoplastic disease contexts are shown to illustrate how recurring OTU-dependent signaling mechanisms translate into intestinal, pulmonary, autoimmune, neuroinflammatory, and metabolic pathology.
