# Supplementary figures and images for: OTU deubiquitinases as immune-circuit editors: from human immunopathology to therapeutic prioritization
Source: Front Immunol. 2026 Jul 1;17:1843621. doi: 10.3389/fimmu.2026.1843621 (PMC13368651; doi:10.3389/fimmu.2026.1843621)

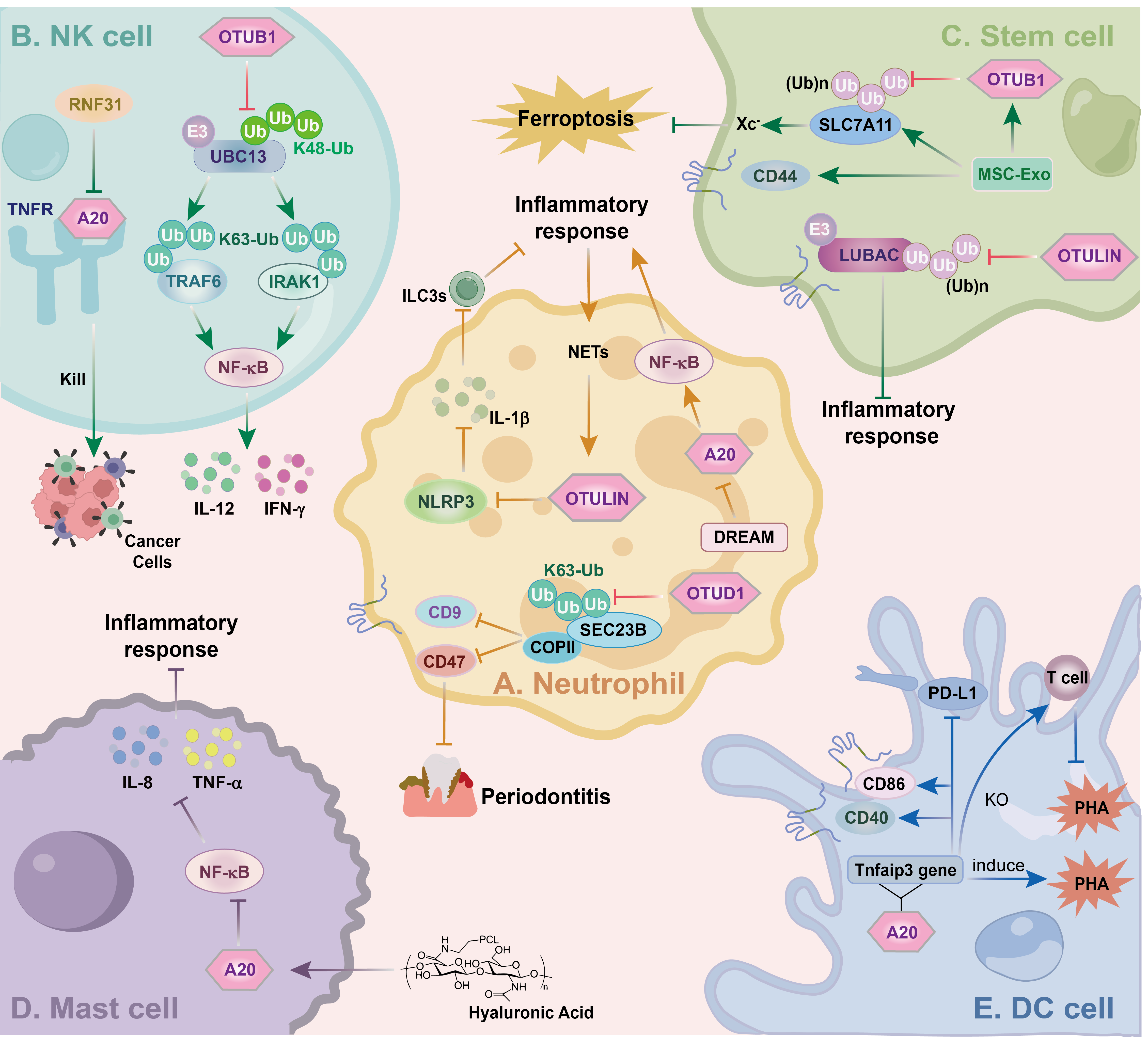

Supplement: Supplementary Figure 1 — Cell-type-specific OTU-dependent regulation in innate immune and stromal-associated contexts. Representative lineage-specific OTU functions in NK cells, neutrophils, mast cells, dendritic cells, and stem-cell-related inflammatory niches are shown to emphasize cell-state-dependent control of inflammatory signaling thresholds. [file Image1.tif]

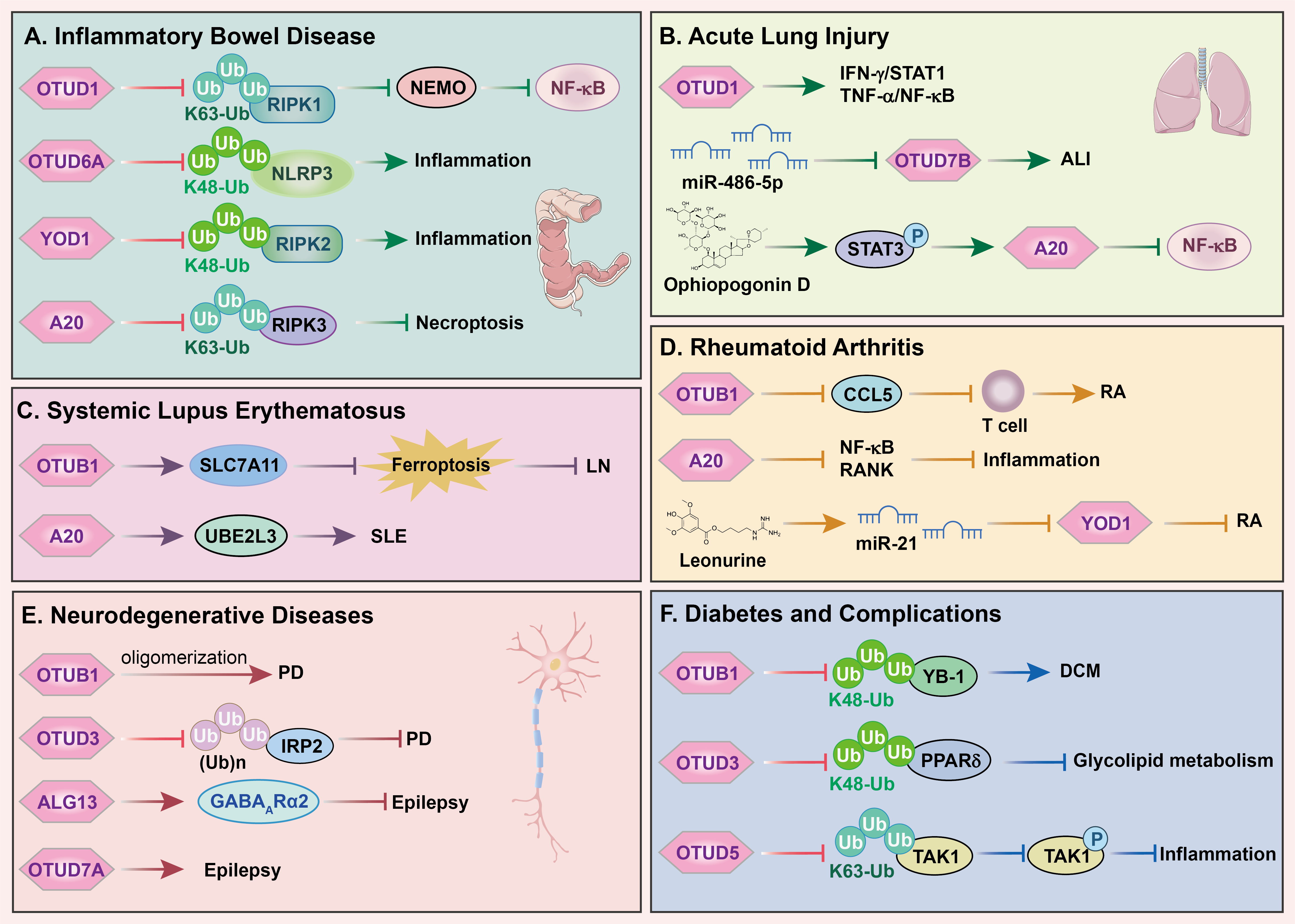

Supplement: Supplementary Figure 2 — Representative non-neoplastic disease contexts associated with OTU dysregulation. Representative non-neoplastic disease contexts are shown to illustrate how recurring OTU-dependent signaling mechanisms translate into intestinal, pulmonary, autoimmune, neuroinflammatory, and metabolic pathology. [file Image2.tif]
